# Ionocytes and CFTR Chloride Channel Expression in Normal and Cystic Fibrosis Nasal and Bronchial Epithelial Cells

**DOI:** 10.3390/cells9092090

**Published:** 2020-09-13

**Authors:** Paolo Scudieri, Ilaria Musante, Arianna Venturini, Daniela Guidone, Michele Genovese, Federico Cresta, Emanuela Caci, Alessandro Palleschi, Marco Poeta, Francesca Santamaria, Fabiana Ciciriello, Vincenzina Lucidi, Luis J. V. Galietta

**Affiliations:** 1 Department of Neurosciences, Rehabilitation, Ophthalmology, Genetics, Maternal and Child Health (DiNOGMI), University of Genova, 16147 Genova, Italy; paolo.scudieri@unige.it (P.S.); ilaria.musante@unige.it (I.M.); 2 Medical Genetics Unit, Istituto Giannina Gaslini, 16147 Genova, Italy; emanuela.caci@unige.it; 3 Telethon Institute of Genetics and Medicine (TIGEM), 80078 Pozzuoli (NA), Italy; a.venturini@tigem.it (A.V.); d.guidone@tigem.it (D.G.); m.genovese@tigem.it (M.G.); 4 Centro Fibrosi Cistica, Istituto Giannina Gaslini, 16147 Genova, Italy; federicocresta@gaslini.org; 5 Thoracic Surgery and Lung Transplantation Unit, Fondazione IRCCS Ca’ Granda-Ospedale Maggiore Policlinico, 20122 Milano, Italy; alepalleschi@gmail.com; 6 Department of Translational Medical Sciences, Università di Napoli “Federico II”, 80131 Napoli, Italy; po3ta.89@gmail.com (M.P.); santamar@unina.it (F.S.); 7 Cystic Fibrosis Unit, Bambino Gesù Children’s Hospital, 00165 Roma, Italy; fabiana.ciciriello@opbg.net (F.C.); vincenzina.lucidi@opbg.net (V.L.)

**Keywords:** CFTR, cystic fibrosis, ionocytes, airway epithelium, chloride secretion

## Abstract

The airway epithelium contains ionocytes, a rare cell type with high expression of Forkhead Box I1 (*FOXI1*) transcription factor and Cystic Fibrosis Transmembrane conductance Regulator (*CFTR*), a chloride channel that is defective in cystic fibrosis (CF). Our aim was to verify if ionocyte development is altered in CF and to investigate the relationship between ionocytes and CFTR-dependent chloride secretion. We collected nasal cells by brushing to determine ionocyte abundance. Nasal and bronchial cells were also expanded in vitro and reprogrammed to differentiated epithelia for morphological and functional studies. We found a relatively high (~3%) ionocyte abundance in ex vivo nasal samples, with no difference between CF and control individuals. In bronchi, ionocytes instead appeared very rarely as previously reported, thus suggesting a possible proximal–distal gradient in human airways. The difference between nasal and bronchial epithelial cells was maintained in culture, which suggests an epigenetic control of ionocyte development. In the differentiation phase of the culture procedure, we used two media that resulted in a different pattern of CFTR expression: confined to ionocytes or more broadly expressed. CFTR function was similar in both conditions, thus indicating that chloride secretion equally occurs irrespective of CFTR expression pattern.

## 1. Introduction

The surface of the airways is covered by an epithelial layer composed of a variety of different cell types [1]. Ciliated cells constitute the most abundant cells in proximal airways. Goblet cells, devoted to the secretion of mucins, are relatively rare, but their number increases as a response to inflammation and infection [2,3,4]. There are also club (secretory) cells, much more frequent in the epithelium of distal airways, that also secrete mucins and other macromolecules with a protective function [5,6]. Continuous regeneration of ciliated, goblet, and secretory cells is provided by basal stem cells in the large airways [7,8,9,10]. In small airways, club cells may have themselves the ability to differentiate into other cell types [11].

The airway epithelium plays an important protective role against noxious agents introduced in the respiratory system through inhaled air [12]. The secretion of mucus strands and threads by goblet cells of the surface epithelium and submucosal glands provides a way to trap these agents [13]. The continuous beating of cilia then propels mucus with entrapped material toward the oropharynx. Productive ciliary beating requires the presence of a fluid that covers the apical (luminal) side of the epithelium [14]. The production of this fluid (airway surface liquid, ASL) involves a fine balance between the secretion and absorption of Cl^−^, Na^+^, HCO_3_^−^, and other ions operated by the coordinated activity of membrane channels and transporters specifically localized on the apical and basolateral membrane of epithelial cells [12,15]. The net transport of electrolytes then drives water by osmosis. Additionally, HCO_3_^−^ may have specific roles in the release and expansion of mucins [16,17,18]. A major pathway for Cl^−^ and HCO_3_^−^ secretion in the airways is provided by Cystic Fibrosis Transmembrane conductance Regulator (CFTR), an anion channel regulated by cAMP [19]. In cystic fibrosis (CF), one of the most frequent genetic diseases, loss of function of CFTR disrupts mucociliary clearance and innate defense mechanisms resulting in airway obstruction by mucus accumulation and bacterial colonization [20,21,22].

Recently, a comprehensive analysis by single-cell RNA sequencing (scRNAseq) investigated the composition of the airway epithelium by grouping cell types on the basis of gene expression similarities [23,24,25]. In addition to well-known cell types (ciliated, goblet, and basal), this analysis revealed the existence of a cell named ionocyte [23,24]. These cells, characterized by a very low abundance (less than 1%), show very high expression of *CFTR* and the transcription factor Forkead Box I1 (FOXI1). Actually, it was found that *CFTR* transcripts in ionocytes represent nearly 50% of total transcripts in the whole epithelium [23,24]. This finding was very intriguing since many previous studies had shown a broader CFTR expression, particularly in ciliated cells [4,26,27]. The precise function of CFTR in ionocytes and whether ionocyte expression in CF is altered are unknown.

In the present study, we investigated the expression of ionocytes in nasal epithelial cells from CF patients and control individuals, upon ex vivo collection and following in vitro culture. Data were compared to those of bronchial epithelial cells. The results showed a relatively high expression of ionocytes in the nasal mucosa, with a possible proximal–distal gradient along the airways, and no difference between CF and non-CF individuals. Interestingly, the difference in ionocyte abundance was also observed in epithelia differentiated from basal cells of nasal and bronchial origin, thus suggesting genetic or epigenetic control of ionocyte expression.

## 2. Materials and Methods

### 2.1. Nasal Brushing Procedure

Control individuals (*n* = 18) and CF patients (*n* = 22) underwent nasal washing with physiological solution (NaCl, 0.9%) in the 12 h preceding the collection. For CF patients, the procedure was carried out in the context of routine outpatient visits already planned for periodic disease control or during hospitalizations for pulmonary exacerbation (PEx) and treatment with IV antibiotics. CF patients affected by active, acute rhinitis at the time of sampling were excluded. For nasal epithelial cell collection, we used the Endobrush^®^ (Biogyn, Mirandola, Italy) cytological sampling brush, consisting of nylon bristles, held by a metal winding and mounted on a plastic stem.

Nasal brushing was performed in both nostrils in every subject involved in the project. The cytological brush was inserted inside nasal cavities in order to brush the mucous membranes of nasal turbinate, by gentle back and forth movements, associated with rotational movements on the axis of the brush itself. The procedure lasted about 4–5 s for each nostril. The brush was then immediately placed in a 15 mL centrifuge tube containing either 10% neutral buffered formalin (05-01005Q; Bio-Optica, Milan, Italy) or culture medium and then transferred to the laboratory for processing. Usually, cells were processed within 24 h after collection. The collection and use of human airway epithelial cells for scientific research was approved by the local Ethical Committee (Comitato Regione Liguria, CER: 28/2020).

### 2.2. Immunofluorescence of Nasal Samples

Upon arrival at the laboratory, the cytological brush carrying fixed cells was sequentially transferred to a 15 mL centrifuge tube containing 10 mL of phosphate-buffered saline (PBS) and then to a 1.5 mL microcentrifuge tube containing 150 µL of PBS. The cells were detached by passing the brush through a 200 µL micropipette tip (with the extreme end removed). Cells detached from the brush were deposited on silanized glass slides placed in a humidified histological chamber. After 2–3 h, cells were processed for immunofluorescence as described previously [4,28,29]. Briefly, after antigen retrieval with 10 mM citrate buffer, the samples were permeabilized with 0.3% Triton X-100 in PBS for 5 min, blocked with 1% bovine serum albumin (BSA) in PBS for 2 h, and then incubated overnight at 4 °C with primary antibodies diluted in PBS containing 1% BSA. The following antibodies and dilutions were used: rabbit anti-FOXI1 (HPA071469; MilliporeSigma, Burlington, MA, USA) at 1:100; mouse IgG1 anti-CFTR (ab570; J.R. Riordan, University of North Carolina at Chapel Hill, Chapel Hill, NC, USA, and Cystic Fibrosis Foundation Therapeutics) at 1:250; mouse IgG1 anti-MUC5AC (MA5-12178; Thermo Fisher Scientific, (Waltham, MA, USA) at 1:200; and mouse IgG2B anti-acetylated tubulin (T7451; MilliporeSigma) at 1:300. Following incubation with primary antibodies, cells were rinsed three times in PBS and incubated with a solution of secondary goat anti-rabbit Alexa Fluor 488, goat anti-mouse IgG1 Alexa Fluor 546, and goat anti-mouse IgG2B Alexa Fluor 633 antibodies (Thermo Fisher Scientific) diluted at 1:200 in PBS containing 1% BSA for 1 h in the dark. After further three washes in PBS, slides were mounted using Fluoroshield with DAPI (MilliporeSigma) to stain cell nuclei.

Confocal microscopy was done using a laser scanning confocal microscope (TCS SPE; Leica Microsystems, Wetzlar, Germany). An image analysis was performed using Leica and ImageJ (NIH) software. For each sample, 400–800 cells were analyzed. To represent the different markers, we chose in each image the best combination of colors. Counting of ionocytes in the different samples was initially done by a single operator who was aware of the identity of the samples. For confirmation, all images were again inspected and counted by a second operator in a blinded way. The results from the two separate procedures were essentially identical. In particular, the significant difference between cultured nasal and bronchial epithelial cells (Figure 4C) was confirmed.

To quantify CFTR expression in the apical membrane of ionocytes, two regions of interest (ROIs) were selected on each FOXI1-positive cell: one on the apical membrane (AM) and another one placed halfway between the apical membrane and the nucleus (C, cytosol). ROI positioning was done in merged fluorescence and bright-field images to easily detect the apical membrane in cells with very low CFTR expression. The mean fluorescence intensity of each ROI was calculated with the ImageJ software, and the AM/C ratio was reported in a dot plot graph.

### 2.3. Anti-FOXI1 Antibody Validation

CFBE41o- cells were cultured in minimal essential medium (MEM; Thermo Fisher Scientific) supplemented with 10% fetal bovine serum (MilliporeSigma), 2 mM l-glutamine, 100 U/mL penicillin, and 100 µg/mL streptomycin. CFBE41o- cells were seeded in a 12-well µ-Chamber (Ibidi, Gräfelfing, Germany) at a density of 25,000 cells/well. After 24 h, cells were transfected with a plasmid carrying the coding sequence for human FOXI1 (a kind gift from Prof. Sven Enerbäck, University of Gothenburg, Gothenburg, Sweden). For each well, 0.2 µg of plasmid DNA and 0.5 µL of Lipofectamine 2000 (Thermo Fisher Scientific) were first premixed in 50 µL of Opti-MEM (Thermo Fisher Scientific) to generate transfection complexes (60 min at room temperature) and were then added to the cells. After 24 h, the complexes were removed by replacement with fresh culture medium. After further 24 h, cells were fixed and processed for immunofluorescence experiments as described for nasal samples.

Image acquisition and analysis were performed using a laser scanning confocal microscope (TCS SPE; Leica Microsystems) and ImageJ software, respectively.

### 2.4. Histological Sections

Bronchial samples obtained from 16 patients undergoing lung transplant (10 CF and 6 non-CF), and bronchial epithelia differentiated in vitro on Snapwell supports were fixed in 10% neutral buffered formalin (05-01005Q; Bio-Optica), embedded in paraffin, and sectioned at 7 µm. The immunofluorescence experiments were carried out as described for nasal samples. To determine the percentage of ionocytes (CFTR-expressing cells) in each image, we counted the cells that showed staining for CFTR in the apical membrane and the total number of nuclei.

### 2.5. Bronchial and Nasal Cell Culture

The procedures for the isolation and culture of human bronchial epithelial cells were described in detail in previous studies [4,28,29]. For nasal epithelial cell culture, after brushing of the nasal mucosa, the cytological brush was immersed in a 15 mL centrifuge tube containing transport medium (RPMI 1640 medium supplemented with 3% penicillin–streptomycin) and delivered to the laboratory. Nasal epithelial cells were collected as described in the immunofluorescence protocol and then seeded in flasks.

Both bronchial and nasal epithelial cells were cultured in a serum-free medium (LHC basal medium/RPMI 1640; Thermo Fisher Scientific) supplemented with various hormones and supplements. The detailed preparation of this medium was previously described (4). To further promote the proliferation of basal stem cells [30], we included a cocktail of compounds consisting of a bone morphogenetic protein (BMP) antagonist (DMH-1; Tocris, Abingdon, UK), a transforming growth factor- β (TGF-β) antagonist (A 83-01; Tocris), and a rho-associated protein kinase 1 (ROCK1) inhibitor (Y-27632; Tocris). After 3–4 passages, cells were seeded at high density (500,000/cm^2^) on Snapwell (3801, Corning Costar) porous inserts. After 24 h from seeding, the proliferative medium was switched to differentiation medium to induce mucociliary differentiation. We used two different differentiation media: BMIX, which we had previously used in several studies [4,28,29,31], and PneumaCult ALI (Stemcell Technologies, Vancouver, Canada). Epithelia differentiated in BMIX were kept under submerged conditions (liquid-liquid interface) for 7 days. Subsequently, the apical medium was totally removed and the cells received nutrients only from the basolateral side (air–liquid interface condition) for further 2 weeks. Instead, epithelia differentiated with PneumaCult ALI medium were directly kept under the air–liquid interface for 2–3 weeks.

### 2.6. Short-Circuit Current Recordings

Snapwell inserts carrying differentiated bronchial epithelia were mounted in a vertical chamber resembling an Ussing system with internal fluid circulation. Both apical and basolateral hemichambers were filled with 5 mL of Krebs bicarbonate solution containing (in mM): 126 NaCl, 0.38 KH_2_PO_4_, 2.13 K_2_HPO_4_, 1 MgSO_4_, 1 CaCl_2_, 24 NaHCO_3_, and 10 glucose. Both sides were continuously bubbled with a gas mixture containing 5% CO_2_ and 95% air, and the temperature of the solution was kept at 37 °C. The transepithelial voltage was short-circuited with a voltage clamp (EVC-4000; World Precision Instruments, Sarasota, FL, USA) connected to the apical and basolateral chambers via Ag/AgCl electrodes and agar bridges (1 M KCl in 2% agar). The offset between voltage electrodes and the fluid resistance was cancelled before experiments. The short-circuit current was recorded with a PowerLab 4/25 (ADInstruments, Dunedin, NewZealand) analog-to-digital converter connected to a computer.

### 2.7. Statistics

Data were presented as representative images/traces or as dot plot graphs. A statistical analysis of data was performed with Student’s *t*-test.

## 3. Results

**Figure 1:**
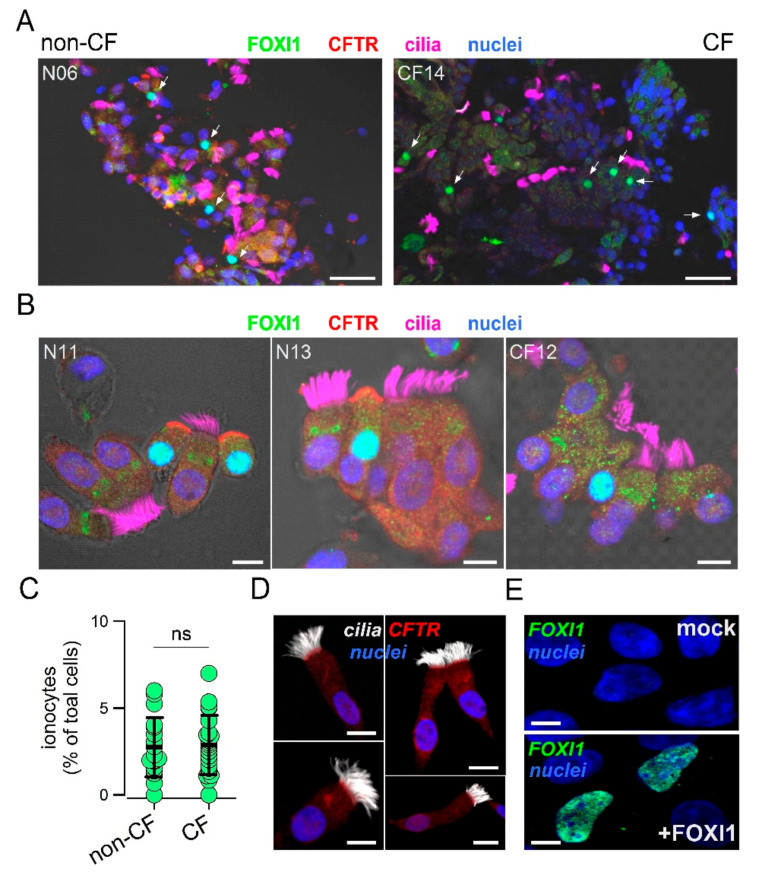
Detection of ionocytes in the human nasal epithelium. (**A**) Representative low magnification images showing staining for CFTR, cilia, FOXI1, and nuclei. The two images are from a control individual and a cystic fibrosis (CF) patient. Arrows indicate nuclei positive for FOXI1. Scale bar: 25 µm. (**B**) High magnification images for two control individuals and one CF patient. Ionocytes were clearly identified in non-CF samples as non-ciliated cells with apical positivity for CFTR and nuclear positivity for FOXI1. In most CF samples, CFTR could not be detected due to mutations impairing protein synthesis or trafficking. Scale bar: 5 µm. (**C**) Abundance of ionocytes in non-CF vs. non-CF samples. Each dot represents the mean percentage of ionocytes over total cells in a single individual (*n* = 18 and 22 for non-CF and CF samples, respectively). (**D**) Representative images from a nasal sample devoid of ionocytes and with CFTR expressed in ciliated cells. Scale bar: 5 µm. (**E**) Validation of the FOXI1 antibody. Images show nuclear signal only in CFBE41o- cells transfected with the FOXI1 plasmid. Scale bar: 5 µm.

To analyze the expression of ionocytes in the nasal epithelium, we devised a simple method to collect cells and preserve their morphology and pattern of protein expression. Epithelial cells were obtained by gentle brushing of nasal mucosa of CF patients and control individuals. After the procedure, the brush was immediately immersed in formalin and transferred from the clinical center to the laboratory. Fixed cells were collected and deposited on silanized glass slides for subsequent immunostaining. The representative low and high magnification images from control and CF samples are shown in Figure 1A,B.

CFTR, FOXI1, and cilia were simultaneously investigated. In non-CF samples, we clearly detected the presence of non-ciliated cells with strong staining for CFTR in the apical membrane and for FOXI1 in the nucleus, a pattern that is consistent with that of ionocytes. At low magnification, ionocyte abundance appeared relatively high since many FOXI1-positive cells were seen within the same field (Figure 1A). In CF nasal samples, FOXI1-positive cells were also easily found (Figure 1A,B). As expected, FOXI1-positive cells in most CF samples were devoid of CFTR staining because many patients carried mutations (F508del, N1303K, G542X, and E585X) that impair the synthesis or normal trafficking of the protein [32]. The scatter dot plot in Figure 1C summarizes the results (percentage of FOXI1-positive cells) obtained for the nasal brushings of 18 non-CF and 22 control individuals. The mean value for CF and non-CF groups was essentially the same and corresponded to nearly 3% of total cells. This abundance of ionocytes was relatively high compared to bronchial epithelia in which a percentage lower than 1% was reported [23,24]. Figure 1D shows a notable exception among all nasal brushing samples. In this non-CF individual, no ionocytes were found and CFTR signal was detected in the apical membrane of ciliated cells. This individual had no pathologies or other conditions that could explain this peculiarity. To validate the results obtained with the FOXI1 antibody, we carried out transfection experiments in CFBE41o- cells. According to gene expression profiling by RNA sequencing, these cells had negligible expression of *FOXI1* gene. A clear nuclear signal appeared by immunofluorescence in cells transfected with the FOXI1 plasmid, but not in mock-transfected cells (Figure 1E).

**Figure 2:**
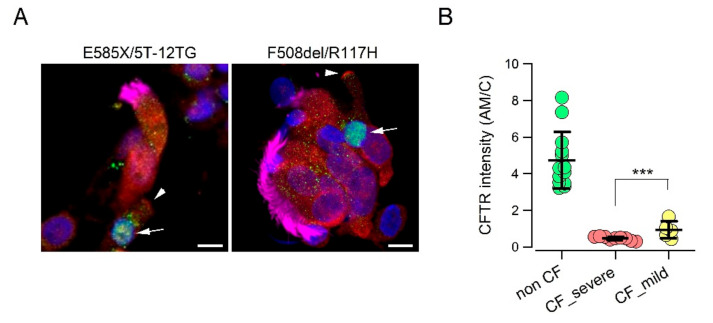
CFTR protein expression in the apical membrane of nasal ionocytes from CF patients with “mild” mutations. (**A**) Representative images of nasal epithelial cells collected by brushing from CF patients with indicated genotypes. Cells were stained for FOXI1 (green), CFTR (red), cilia (magenta), and nuclei (blue). Arrows indicate nuclei positive for FOXI1. Arrowheads indicate CFTR signal in the apical membrane. (**B**) Scatter dot plot reporting the intensity of CFTR signal in the apical membrane (relative to signal in the cytosol, AM/C) of FOXI1-positive cells. This parameter was significantly higher (***, *p* < 0.01) in cells with “mild” mutations compared to cells with severe mutations.

We asked whether a relationship could be established between the *CFTR* genotype and extent of CFTR protein expression in the apical membrane of ionocytes. We compared cells from non-CF individuals with those from two groups of CF patients. The first CF group included patients with “severe” mutations that impair CFTR protein synthesis or trafficking, including F508del, E585X, N1303K, deletions, and frameshift mutations [32,33]. The second group included patients carrying in one allele a “milder” mutation such as D1152H, R117H, or the 5T/12TG polymorphism [28,29]. Interestingly, we were able to detect a CFTR signal in the apical membrane of cells with less severe mutations. Although lower than that of non-CF cells, the signal was significantly higher than that of cells with severe mutations (Figure 2A,B).

The nasal brushing samples used for ionocyte detection were also separately processed to further analyze the composition of the epithelium. In particular, Supplementary Figure S1A shows representative images reporting simultaneous detection of acetylated tubulin and MUC5AC mucin as markers of ciliated and goblet cells, respectively. CF samples showed a significant decrease in ciliated cells compared with control samples, whereas goblet cells were not significantly different (Figure S1B).

**Figure 3:**
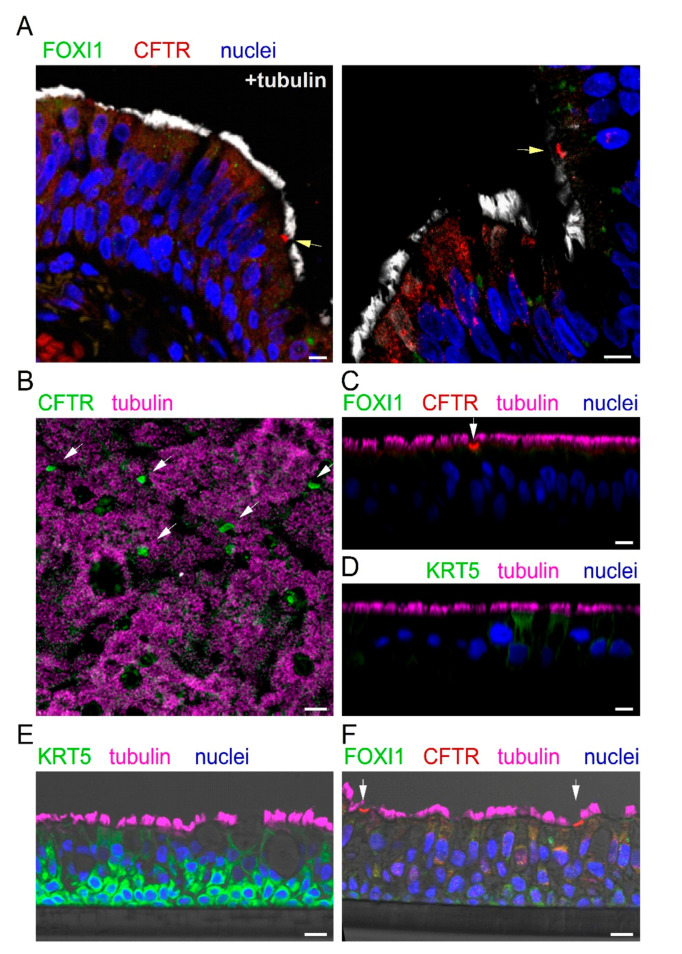
CFTR expression in bronchial epithelium in vivo and in vitro. (**A**) Representative immunofluorescence images of bronchial sections showing staining for CFTR (red), cilia (white), FOXI1 (green), and nuclei (blue). Arrows indicate the presence of putative ionocytes, i.e., non-ciliated cells with strong CFTR positivity in the apical membrane. Nuclear FOXI1 staining was never detected in these histological preparations. (**B**–**D**) Immunofluorescence analysis on cultured bronchial epithelia, after fixation and permeabilization. CFTR was expressed in isolated non-ciliated cells ((**B**), xy scan; (**C**), xz scan). FOXI1 was instead undetectable (**C**). KRT5, a marker of basal cells, was also absent ((**D**), xz scan). (**E**,**F**) Immunofluorescence analysis on cultured bronchial epithelia, after inclusion in paraffin and sectioning. In contrast to intact epithelium, sectioning allowed the detection of KRT5 in basal cells (**E**). In contrast, FOXI1 signal was still absent in CFTR-expressing cells (**F**). Scale bar: 10 µm in (**A**) and (**C**–**F**) and 25 µm in (**B**).

We looked for the expression of ionocytes in the bronchial epithelium. Since collection of cells by bronchial brushings was not feasible, we relied on bronchi resected from explanted lungs at the time of lung transplant of CF and non-CF patients. Bronchial samples were fixed in formalin, embedded in paraffin, and sectioned. Figure 3A shows two representative images reporting the analysis of CFTR, FOXI1, and cilia expression by immunofluorescence. We observed rare non-ciliated cells with marked CFTR staining in the apical membrane (arrow). These cells were always negative for FOXI1. In general, FOXI1 signal was never detected. We considered the CFTR-positive cells as putative ionocytes and quantified their abundance. Ionocytes corresponded to less than 0.5% of total cells, a value that was consistent with that reported by scRNAseq for human bronchial epithelia [23,24,25].

We investigated the expression of ionocytes in cultured bronchial epithelial cells. Cells were detached from bronchi by protease digestion and then expanded in a serum-free medium (LHC9/RPMI 1640). After the proliferation phase, cells were seeded at high density on porous membranes, switching the culture medium to PneumaCult ALI to induce differentiation. This process was also favored by keeping the cells under air–liquid interface (ALI) condition, i.e., with no medium on the apical surface. The resulting epithelia were fixed in formalin; permeabilized; immunostained for CFTR, FOXI1, and cilia; and inspected by laser scanning confocal microscopy. We found a strong CFTR signal in rare non-ciliated cells, but no FOXI1 expression, even by scanning the epithelium in the z-axis (Figure 3B,C).

We were concerned that the lack of FOXI1 staining could be due to the inability of the antibody to penetrate the epithelium. Indeed, epithelia appeared thick, with an average distance between the basal and apical membrane of 45–50 µm. Therefore, we used an antibody against keratine 5 (KRT5), a marker of basal cells. We found no staining for KRT5 (Figure 3D), thus demonstrating that there is indeed a limitation in the diffusion of antibodies from the apical side into the deepest regions of the epithelium. To circumvent this problem, we proceeded in a different way. Cultured epithelia were embedded in paraffin and sectioned to facilitate the penetration of antibodies to all layers of the epithelium. On these samples, we succeeded in detecting KRT5 (Figure 3E). A nice layer of basal cells, strongly positive for KRT5, was observed. However, we failed in detecting FOXI1 on these sections, also in areas of the epithelium that contained cells with strong CFTR staining (Figure 3F).

**Figure 4:**
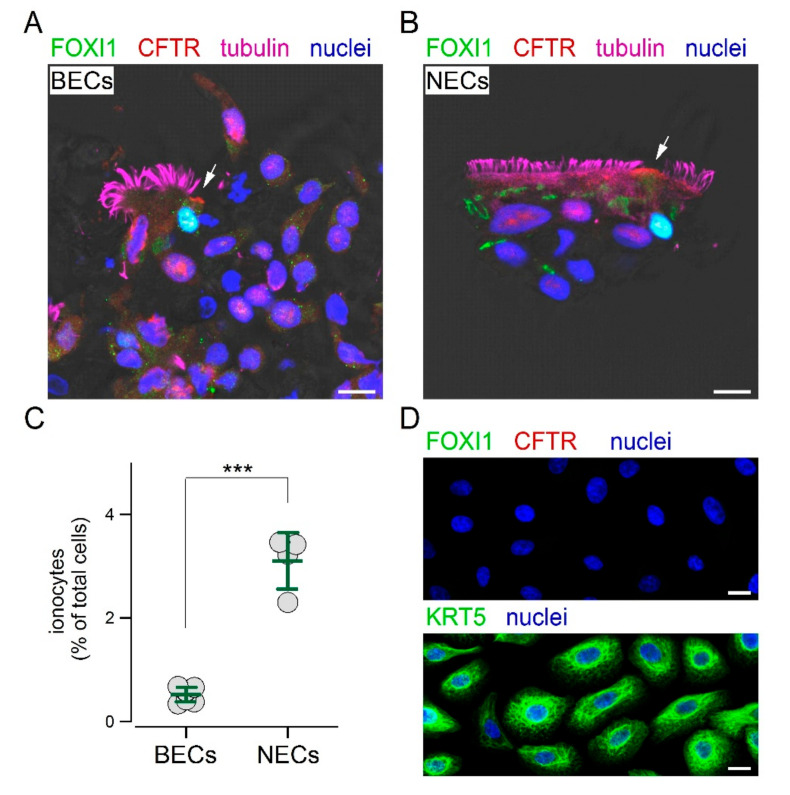
Detection of ionocytes in cultured epithelial cells. (**A**,**B**) Immunofluorescence analysis of cultured bronchial (BECs) and nasal (NECs) epithelial cells after brushing. Under this condition, FOXI1 and CFTR were detected within the same non-ciliated cells. Arrows indicate CFTR signal in the apical membrane. Scale bar: 10 µm. (**C**) Abundance of ionocytes in cultured BECs and NECs. The percentage of ionocytes was significantly (***, *p* < 0.001) higher in NECs. (**D**) Immunofluorescence analysis of airway epithelial cells in the expansion phase of the culture procedure. These cells expressed the basal cell marker KRT5, but not CFTR or FOXI1. Scale bar: 5 µm.

We reasoned that the inclusion in paraffin could be a step preventing the access of the FOXI1 antibody to its target. Therefore, we mechanically detached the cells by brushing the epithelium and then applied the same fixation and immunostaining procedures used for nasal sample collection. With this approach, we finally succeeded in detecting ionocytes in cultured bronchial epithelial cells. Figure 4A shows the presence of non-ciliated cells with both CFTR and FOXI1 expression. The same approach was also successfully used for cultured nasal epithelial cells (Figure 4B). The scatter dot plot in Figure 4C compares the percentage of ionocytes in cultured bronchial and nasal epithelial cells. Interestingly, we found a significantly higher abundance of ionocytes in epithelia derived from nasal mucosa. We asked whether these ionocytes were a remainder of the original epithelium in vivo that persisted in the culture, or were regenerated during the re-differentiation process in vitro. Therefore, we investigated the composition of epithelial cells during the proliferation phase on a solid support. These cells were prevalently positive for KRT5, but showed no FOXI1 expression (Figure 4D). We also looked for other proteins. Proliferating cells were also positive for p63, another basal cell marker, but not for CFTR, cilia, and MUC5AC (Figure S2).

**Figure 5:**
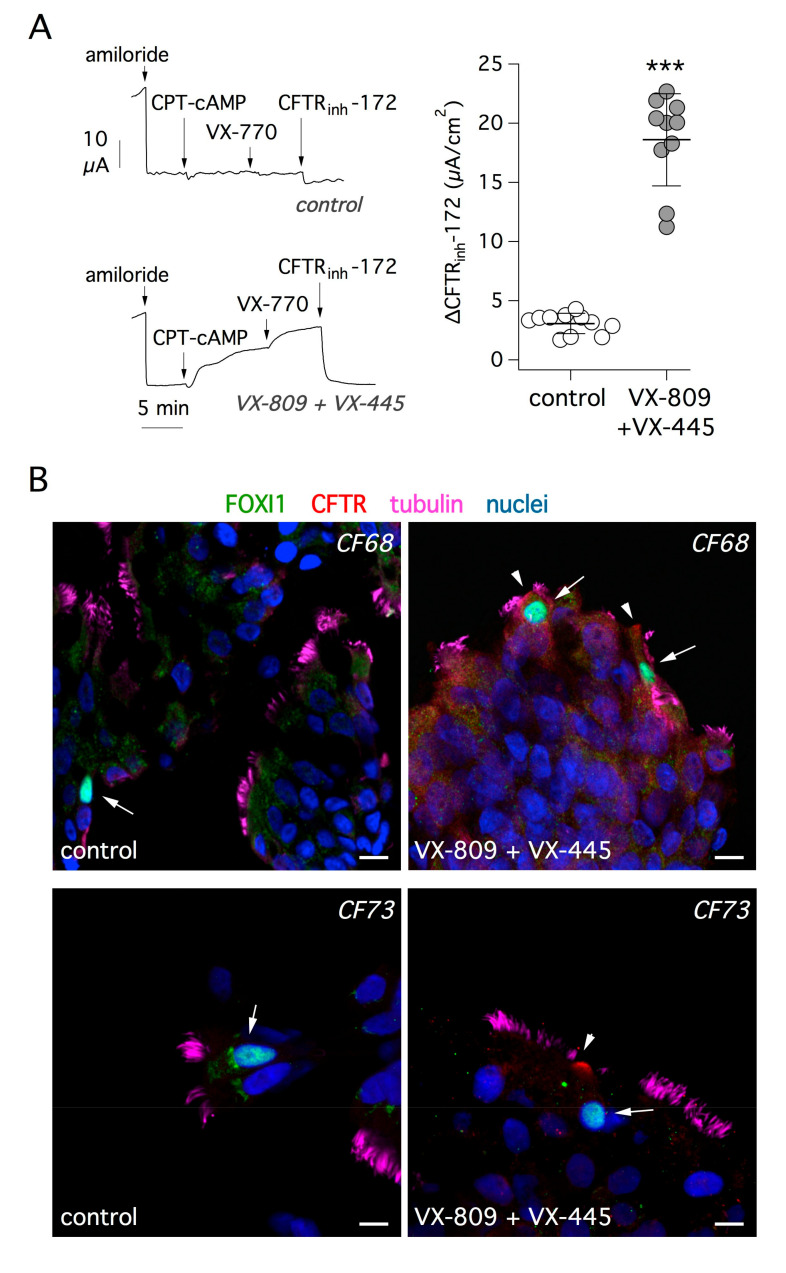
Rescue of F508del-CFTR function and trafficking by a combination of correctors. (**A**) Representative short-circuit current recordings (left) and summary of results (right) from experiments on CF bronchial epithelial cells (F508del/F508del genotype). Cells were treated for 24 h with vehicle alone or with a combination of correctors: VX-809 (1 µM) plus VX-445 (5 µM). During experiments, the epithelial Na^+^ channel (ENaC) was blocked with amiloride (10 µM). CFTR-dependent Cl^−^ secretion was then stimulated with 8-(4-chlorophenylthio)adenosine 3′,5′-cyclic monophosphate (CPT-cAMP, 100 µM) and the potentiator VX-770 (1 µM). Finally, CFTR activity was inhibited with CFTR_inh_-172 (10 µM). The scatter dot plot on the right reports the amplitude of CFTR_inh_-172 effect, which reflects the extent of CFTR function. The results demonstrate significant (***, *p* < 0.001) rescue of F508del-CFTR by the combination of correctors. (**B**) Analysis of F508del-CFTR protein rescue by immunofluorescence. The figure shows representative immunofluorescence images of cultured bronchial epithelial cells from two CF patients (CF68 and CF73). Cells were treated with vehicle (control, left) or with VX-809 plus VX-445 (right) and then stained for FOXI1 (arrows), CFTR, tubulin (cilia), and nuclei. The treatment with correctors led to the appearance of CFTR signal in the apical membrane (arrowheads). Scale bar: 10 µm.

The basic defect caused by F508del mutation, the most frequent among CF patients, can be highly corrected with a combination of small molecules called “correctors”, which act by improving the stability of CFTR protein and its trafficking to the plasma membrane [34,35,36]. We asked whether treatment with correctors is effective in rescuing mutant CFTR protein in ionocytes. Short-circuit current recordings depicted in Figure 5A show the effect of treating bronchial epithelial cells from F508del/F508del patients with VX-809 and VX-445 correctors for 24 h. CFTR activity was acutely stimulated with a membrane-permeable cAMP analog and further enhanced with the VX-770 potentiator. As expected, the extent of CFTR function was markedly increased in cells treated with the corrector combination, compared with cells treated with vehicle alone. This was also evident from the amplitude of the response to the CFTR inhibitor, CFTR_inh_-172, which underwent a six-fold increase with corrector treatment (Figure 5A).

Given the high efficacy of the corrector combination, we looked for evidence of improved trafficking of mutant CFTR protein. Figure 5B shows representative images of cells investigated by immunofluorescence after brushing of cultured epithelia. After treatment with correctors, FOXI1-positive cells showed the appearance of CFTR signal in the apical membrane. Supplementary Figure S3 shows additional images of control and corrector-treated cells. We applied the analysis of CFTR signal in the apical membrane relative to cytosol (AM/C) as explained for Figure 2. We found a significant increase of apical CFTR signal after treatment (Figure S3).

**Figure 6:**
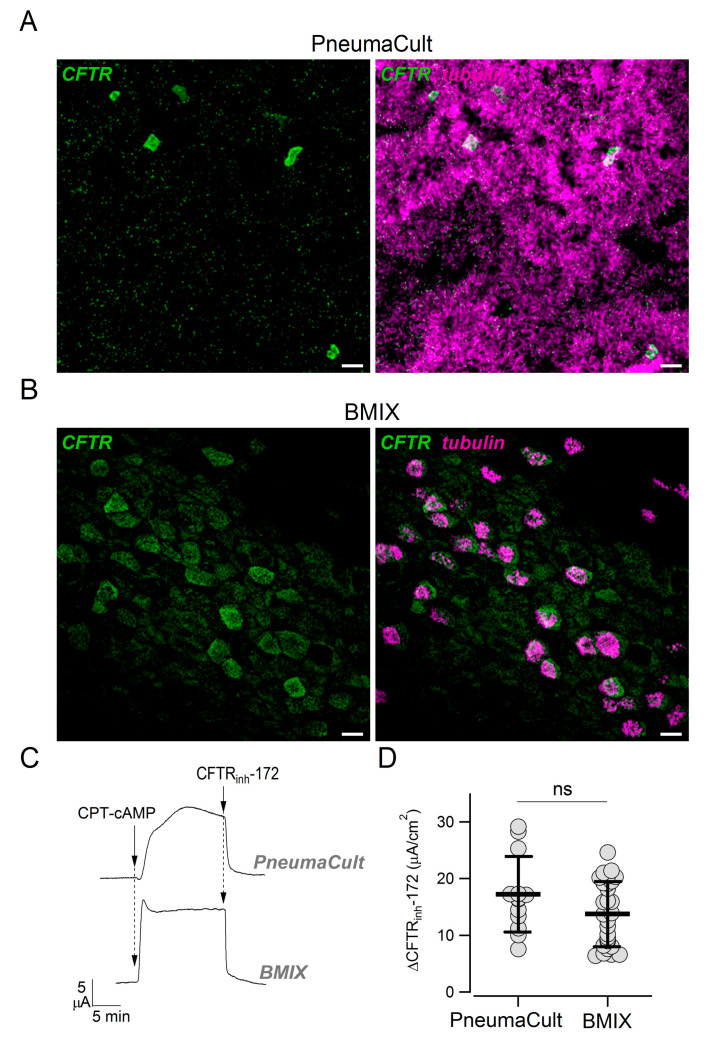
Effect of culture medium on CFTR expression pattern. (**A**,**B**) Detection of CFTR and cilia in epithelia differentiated in PneumaCult ALI or BMIX medium. Scale bar: 10 µm. (**C**) Representative short-circuit current recordings showing activation (CPT-cAMP, 100 µM) and inhibition (CFTR_inh_-172, 10 µM) of CFTR in bronchial epithelia generated with PneumaCult ALI or BMIX. (**D**) CFTR activity measured in PneumaCult ALI and BMIX epithelia. The plot reports the size of the effect of CFTR_inh_-172 after maximal activation of CFTR with CPT-cAMP. The two groups of data were not significantly different.

In previous studies, we did not notice a pattern of CFTR expression suggestive of the presence of ionocytes, i.e., strong CFTR expression in rare non-ciliated cells [4,28]. Actually, CFTR appeared more broadly expressed, particularly in ciliated cells. We asked whether those results were influenced by the culture medium (BMIX) used in our previous studies. Therefore, we compared side-by-side epithelia kept in the two cultured media. Epithelia kept in PneumaCult ALI were highly ciliated, with multiple layers of cells, and showed the presence of rare non-ciliated cells with high CFTR positivity (Figure 6A). In contrast, epithelia kept in BMIX had a lower number of ciliated cells, were mostly composed of a monolayer of cells, and showed a more diffuse CFTR expression (Figure 6B). Interestingly, short-circuit current experiments done on these two types of epithelia revealed a similar amplitude of CFTR currents (Figure 6C,D).

## 4. Discussion

Recently, ionocytes were found in the human airway epithelium as a rare cell type characterized by high expression of CFTR, the Cl^−^ channel that is defective in CF [23,24]. We asked whether the number of ionocytes is altered in CF airways. Actually, it could be hypothesized that an increase in ionocytes occurs in CF as an attempt to compensate for the lack of CFTR-dependent Cl^−^ secretion. To assess ionocyte abundance, we collected nasal epithelial cells and developed a simple protocol allowing the investigation of cell morphology and the expression of cell type-specific markers. Interestingly, we found that ionocytes (identified by the lack of cilia, by strong CFTR staining in the apical membrane, and selective FOXI1 expression in the cell nucleus) were quite abundant in the nasal epithelium in vivo. On average, the percentage of ionocytes was relatively high (nearly 3%) compared to data published on the bronchial epithelium [23,24]. Furthermore, we found that the percentage of ionocytes (i.e., FOXI1-positive cells) in the nasal epithelium of CF patients was not different from that of control individuals. Therefore, lack of functional CFTR does not seem to affect the abundance of ionocytes. We also investigated the extent of CFTR protein expression in the apical membrane of CF ionocytes. CFTR signal was essentially absent in cells from patients with “severe” mutations that cause block of CFTR protein synthesis or trafficking (e.g., F508del or E585X). Instead, we detected CFTR in the apical membrane of nasal ionocytes from patients with relatively “mild” mutations such as R117H or D1152H. This finding suggests that this type of analysis could be useful in future studies to investigate genotype–phenotype correlation and, possibly, the efficacy of CFTR pharmacotherapies. In this respect, our experiments on cultured cells suggest that rescue of F508del-CFTR at the functional level is paralleled by appearance of CFTR signal in the apical membrane of ionocytes.

We further investigated the expression of ionocytes in the bronchial epithelium in vivo to compare the results with those previously published [23,24]. We found very rare non-ciliated cells with strong CFTR staining. Although, we could not detect FOXI1 in these samples, probably due to the paraffin inclusion step, we assumed that these cells correspond to ionocytes. The percentage of ionocytes (less than 0.5%) in our bronchial samples appeared to be lower than that estimated in the nasal epithelium and similar to the value published in previous reports [23,24].

To further investigate the difference between nasal and bronchial epithelial cells, we used cell cultures. In our initial experiments on cultured cells, we detected putative ionocytes, non-ciliated cells with strong CFTR signal, but without FOXI1 expression. However, the lack of FOXI1 detection was related to the processing of the samples. In fixed-permeabilized epithelia, antibodies could not penetrate deeply into the sample as demonstrated by the inability to stain basal cells with antibodies against KRT5. In sections of cultured epithelia, KRT5 expression was clearly visible, but FOXI1 was again undetectable, probably because of embedding in paraffin. Indeed, when we “brushed” cultured epithelia and used the same procedure used for nasal brushing, we were finally successful in detecting FOXI1 and in conclusively identifying ionocytes. Interestingly, we found that ionocytes were significantly more abundant in epithelia of nasal origin, five-fold more than that in cultured bronchial epithelia. We asked whether this difference arose from a persistence of nasal ionocytes during the collection and then expansion of epithelial cells. Therefore, we looked for FOXI1 expression in epithelial cells during the proliferative phase that precedes the differentiation on porous membranes. These cells were completely devoid of FOXI1 expression, as well as of CFTR, cilia, and MUC5AC. The lack of FOXI1 signal was not due to the fixation–staining procedure since we could clearly detect FOXI1 in cells transfected with the corresponding plasmid. Proliferating cells were instead markedly positive for KRT5 and p63, i.e., markers of basal cells. Therefore, the cells that are expanded in the proliferation phase act as stem cells that generate all other cell types, including ionocytes. Accordingly, a specific program, based on genetic or epigenetic mechanisms, is responsible for the different expression of ionocytes in nasal-derived vs. bronchial-derived cell cultures.

In previous studies, we reported a more diffuse expression of CFTR [4,28]. Those studies were carried out with a different culture medium called BMIX. We compared the properties of epithelia kept in PneumaCult ALI with those kept in BMIX. The morphology of epithelium and the pattern of CFTR expression were clearly different. In particular, CFTR was more broadly expressed in the BMIX medium. Since epithelia differentiated with BMIX are less columnar, less ciliated, and with high expression of SCGB1A1 [28], we can speculate that they are more similar to the epithelium present in small airways. In contrast, epithelia differentiated with PneumaCult ALI (columnar cells, predominantly ciliated) are more representative of proximal airways. The factors in the two media that are responsible for these morphological differences are unclear. While BMIX is a home-made medium with a known composition of different hormones and supplements, PneumaCult ALI is a commercial product with proprietary formulation. It would be important in future studies to understand the factors that guide epithelial differentiation.

We compared CFTR function in epithelia generated with the two culture media and surprisingly found no significant difference, suggesting that CFTR-dependent Cl^−^ secretion may equally occur in epithelia where CFTR is confined to a few cells and in epithelia with a more diffuse CFTR localization. Two different hypotheses can be formulated to explain this intriguing finding. It is possible that confinement of CFTR to a small number of cells, namely ionocytes, occurs together with specific upregulation in the same cells of channels and transporters that provide the driving force required for Cl^−^ exit at the apical membrane [12,15]. In other words, CFTR-dependent Cl^−^ secretion is mainly mediated by ionocytes when these cells are present. Alternatively, it can be hypothesized that high CFTR expression in ionocytes is devoted to another as-yet-unknown function. If so, Cl^−^ transport could be mediated by the other cell types (particularly ciliated) in which CFTR expression is low, hardly detectable at the mRNA and protein levels with available methods, but still sufficient to sustain transepithelial Cl^−^ secretion.

The reason for a variable organization of CFTR expression in the airway epithelium is presently unclear. It may be taken into account that we found a notable exception among all nasal samples, consisting of a brushing that showed no ionocytes at all and expression of CFTR in ciliated cells. This finding could suggest that under particular circumstances or in specific individuals the pattern of CFTR expression is altered. Future studies will need to establish if the pattern of CFTR expression is affected by inter-individual differences or by endogenous or environmental stimuli.

In conclusion, our study revealed that ionocytes are particularly expressed in the nasal epithelium and suggested the existence of a proximal-distal gradient with a progressively decreasing number of ionocytes. Accordingly, in more distal regions, CFTR would be expressed in other cell types. The reason for the restricted CFTR expression in a small number of cells in proximal airways is unclear. We can speculate that this pattern is related to higher exposure of these airway regions to challenges coming from the environment such as abrupt changes in temperature and moisture. Further investigation of the mechanisms controlling ionocyte generation and behavior will be essential to further understand the role of the airway epithelium.

## Supplementary Materials

The following are available online at https://www.mdpi.com/2073-4409/9/9/2090/s1, Figure S1: Analysis of nasal epithelium composition, Figure S2: Characterization of cells in the expansion phase, Figure S3: Rescue of F508del-CFTR expression by combination of correctors.
