# Supplementary material for: Ionocytes and CFTR Chloride Channel Expression in Normal and Cystic Fibrosis Nasal and Bronchial Epithelial Cells
Source: Cells. 2020 Sep 13;9(9):2090. doi: 10.3390/cells9092090 (PMC7565890; doi:10.3390/cells9092090)
Supplement: Supplementary file 1 [file cells-09-02090-s001.pdf]

SUPPLEMENTARY MATERIAL

IONOCYTES AND CFTR CHLORIDE CHANNEL EXPRESSION IN NORMAL AND CYSTIC FIBROSIS NASAL AND BRONCHIAL EPITHELIAL CELLS

Paolo Scudieri, Ilaria Musante, Arianna Venturini, Daniela Guidone, Michele Genovese, Federico Cresta, Emanuela Caci, Alessandro Palleschi, Marco Poeta, Francesca Santamaria, Fabiana Ciciriello, Vincenzina Lucidi, Luis J.V. Galietta

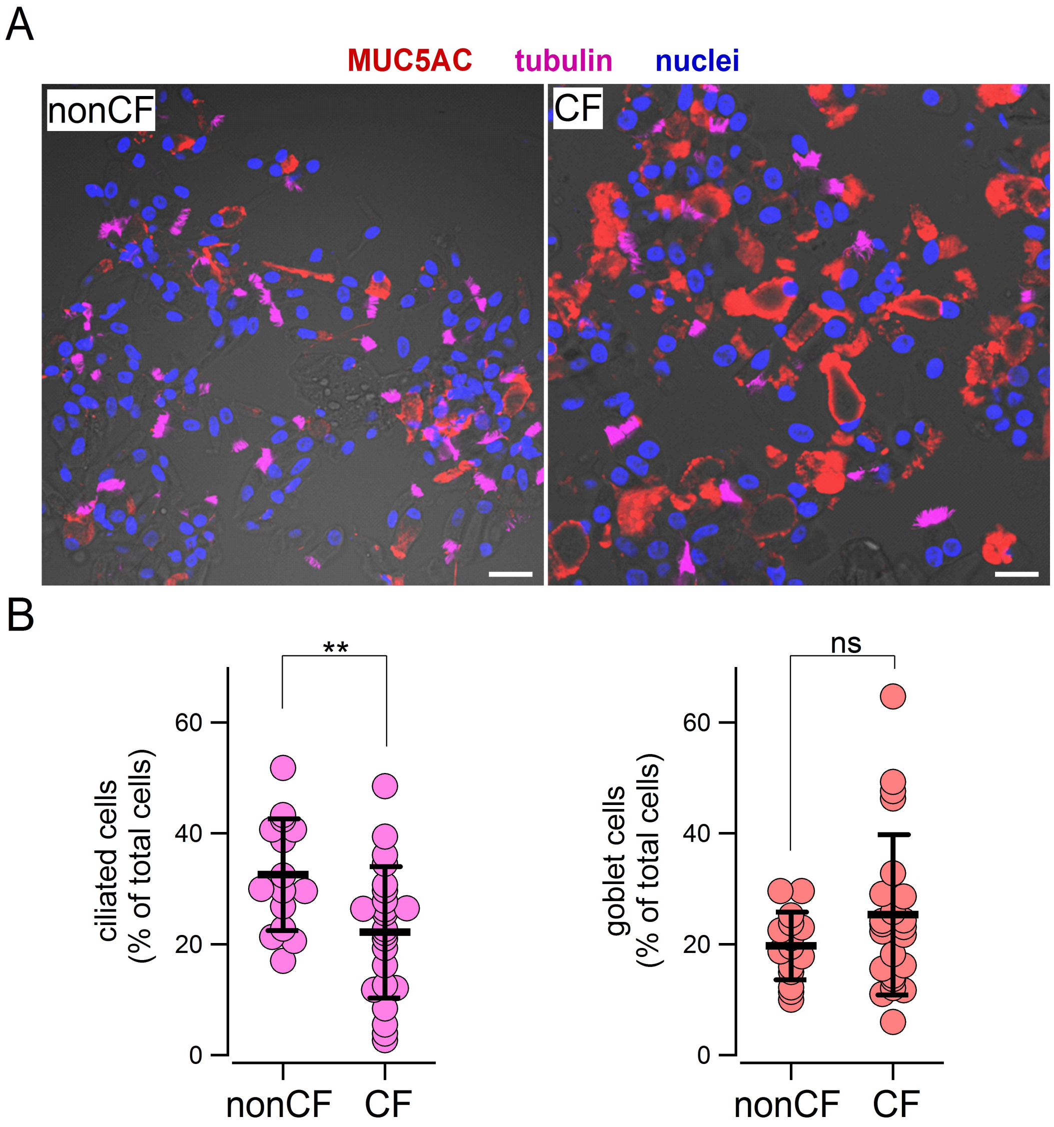

Supplementary Figure 1. Analysis of nasal epithelium composition. (A) Representative images showing detection of MUC5AC mucin and acetylated tubulin as markers of goblet and ciliated cells, respectively. Scale bar: 25 µm. (B) Scatter dot plots reporting the percentage of ciliated (left) and goblet (right) cells in non-CF and CF samples. A significantly ($p < 0.01$) lower number of ciliated cells was found in CF vs. non-CF samples. The number of goblet cells in the two groups of samples was not significantly different.

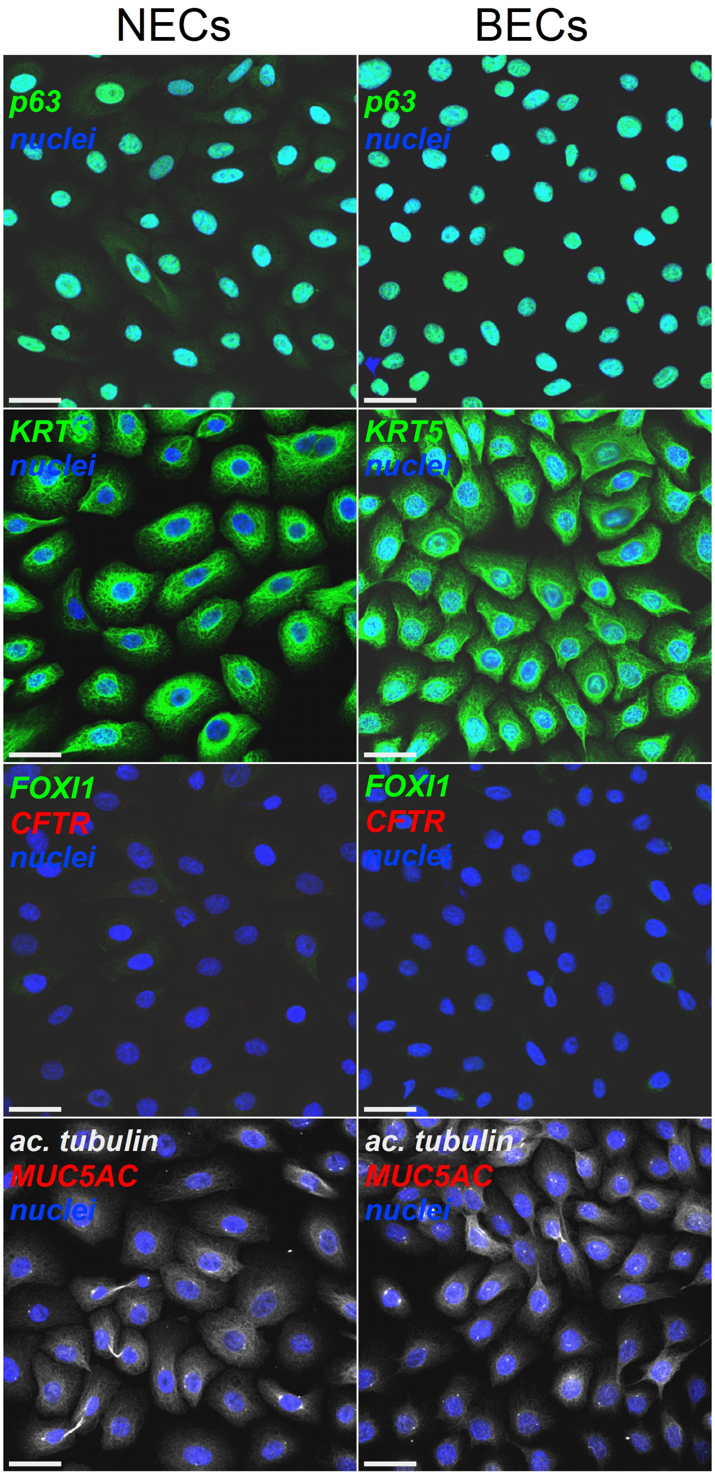

Supplementary Figure 2. Characterization of cells in the expansion phase. The different images show immunofluorescence analysis of nasal (NECs) and bronchial (BECs) epithelial cells during the expansion phase of the culture procedure. The expression of the following markers was investigated: p63, KRT5, FOXI1, CFTR, acetylated tubulin, and MUC5AC. KRT5 and p63 were the only markers expressed, in agreement with proliferating cells corresponding to basal stem cells. Scale bar: 10 µm.

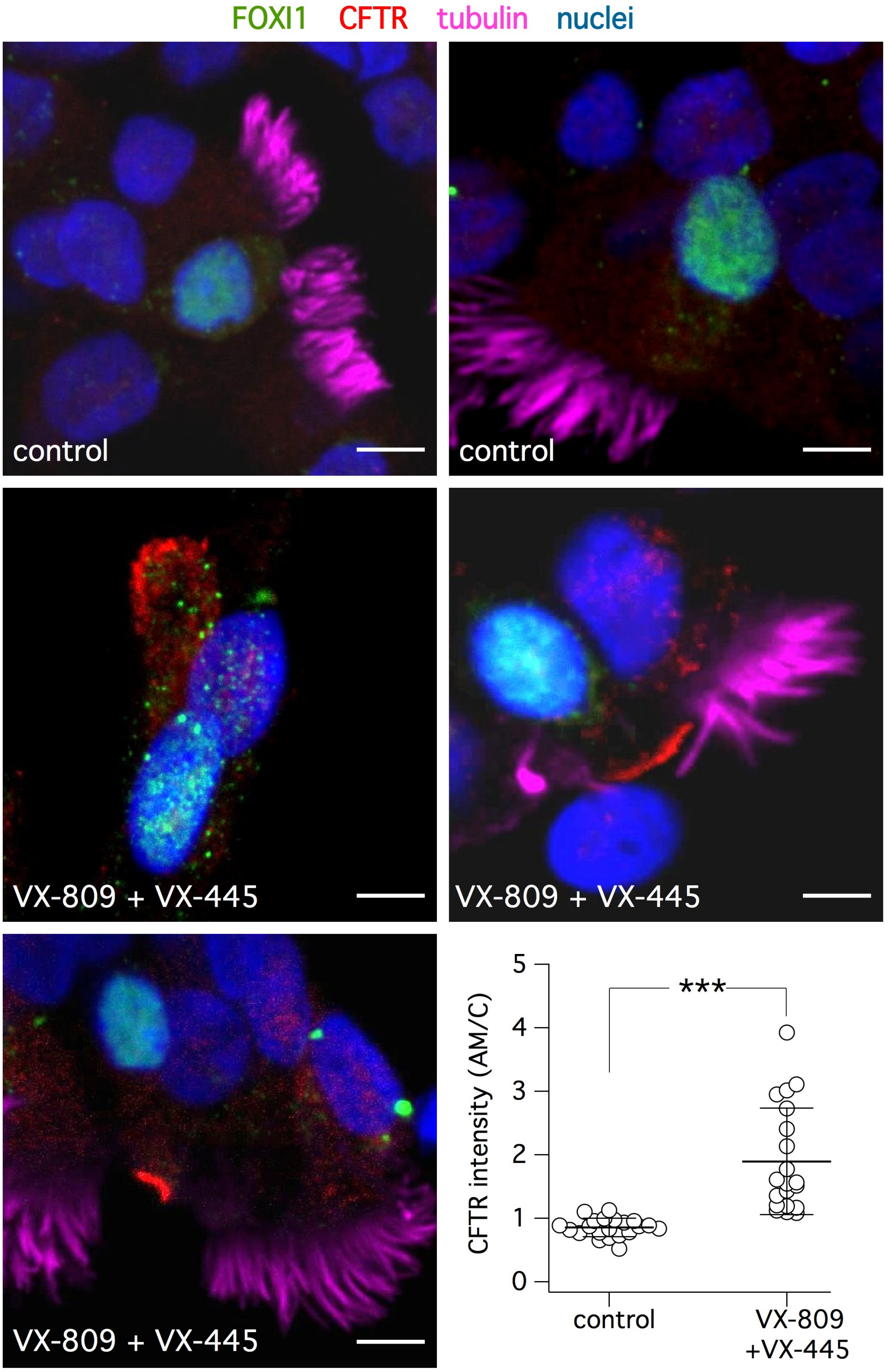

Supplementary Figure 3. Rescue of F508del-CFTR expression by combination of correctors. Representative immunofluorescence images and scatter dot plot reporting the result of cell treatment with vehicle (control) or with the combination of VX-809 (1 µM) and VX-445 (5 µM) for 24 hours. Each image shows detection of FOXI1, CFTR, tubulin (cilia), and nuclei. Scale bar: 5 µm. Treatment with the combination of correctors leads to the appearance of CFTR in the apical membrane. The graph reports the intensity of CFTR in the apical membrane (relative to signal in the cytosol, AM/C) of FOXI1-positive cells. The difference between the two groups of cells was significantly different (***, $p < 0.001$).
